# Higher biosecurity level was associated with reduced risk of Danish dairy cattle farms becoming test-positive for *Salmonella* Dublin in a nested case–control study

**DOI:** 10.3389/fvets.2025.1566380

**Published:** 2025-05-19

**Authors:** Lars Pedersen, Hans Houe, Erik Rattenborg, Liza Rosenbaum Nielsen

**Affiliations:** ^1^Department of Veterinary and Animal Sciences, Section for Animal Health and Welfare, University of Copenhagen, Frederiksberg, Denmark; ^2^SEGES Innovation P/S, Animal Health and Welfare, Cattle Livestock, Aarhus, Denmark

**Keywords:** biosecurity, *Salmonella*, one health, control, prevention, cattle, dairy herds

## Abstract

*Salmonella* Dublin (*S*. Dublin) is a cattle-adapted bacterium with enzootic occurrence in cattle populations of many countries. Preventing the spread of *S.* Dublin between cattle farms requires an understanding of the local pathways for the direct and indirect transmission of bacteria. Identifying key risk factors is complicated due to the numerous pathways through which the bacteria can be introduced and established on dairy cattle farms. This study aimed to provide new knowledge about the effect of biosecurity in dairy farms in *S.* Dublin-enzootic areas of Denmark. The association between the researcher-assessed biosecurity level and the risk of introducing and establishing *S.* Dublin in farms was investigated by following a monthly recalculated cohort of dairy farms with no test-positive *S.* Dublin surveillance results over the previous 2 years. There were 37 new test-positive farms matched by herd size with 74 control farms that remained test negative in the mandatory *S.* Dublin surveillance programme. A published Biosecurity Assessment Framework for *S.* Dublin (BAF-SD) was used to systematically and semi-quantitatively assess the on-farm biosecurity practices across 12 farm sections. Each section was scored on a scale from 0 (total lack of biosecurity measures) to 100 (excellent biosecurity) based on observations and interviews. Lower biosecurity scores in the sections” entrance area,” “pick-up-delivery of calves,” “calves < 130 days,” “cattle > 130 days,” and “storage of feed and feeding” were associated with becoming test-positive for *S*. Dublin at a 90% confidence interval (CI) level in univariable logistic analyses. In the multivariable analysis, a higher weighted biosecurity score across all sections was found to be associated with (*p* < 0.05) with lower odds of becoming test-positive for *S.* Dublin (odds ratio [OR] = 0.64 per 10-unit increase in biosecurity level). None of the study farms had very good (score 80 to <90) or excellent biosecurity (score of 90 or above), highlighting the opportunities for biosecurity improvements on-farm. In conclusion, the current biosecurity levels in Danish farms appear insufficient to resist the infection pressure of *S.* Dublin from the farm surroundings. Hence, biosecurity practices need to be improved, and/or the infection pressure needs to be reduced, to lower the number of new test-positive dairy cattle farms in Denmark.

## 1 Introduction

*Salmonella* Dublin, a bacterium that is host-adapted to cattle (1, 2), causes losses for the dairy industry (3–6) and is a serious zoonotic hazard (7, 8). Among European cattle, *S.* Dublin is the most frequently reported *Salmonella* serotype, with a reported prevalence of up to 40% test-positive dairy farms in some countries (9, 10). In the late 1990s, Denmark initiated monitoring for *S.* Dublin on cattle farms, which in 2008 evolved into a national control programme aimed at eradicating *S*. Dublin from the Danish cattle population (11, 12). The prevalence of “likely infected” dairy cattle farms declined from above 25 to 7.1% in December 2015 (12, 13). Since then, the prevalence has steadily increased to above 10% in December 2021. Despite progressively tighter biosecurity control measures (including strictly regulated movement of animals out of ‘likely infected’ farms for live purposes), new farms continue to become infected or re-infected, especially in enzootic areas (14–19). This suggests that local direct or indirect transmission pathways drive the S. Dublin enzootic occurrence, and a better understanding of these pathways is required to more effectively control the disease. Indeed, with the excretion of *S.* Dublin in faeces and its ability to survive for weeks in slurry and even for years in dried manure (20, 21), transmission by fomites is a likely source of introduction and establishment of *S.* Dublin in cattle farms. Furthermore, the dairy sector is undergoing structural development toward larger, and more complex (multi-site) farms. This increases the frequency of exposure risks through local transmission pathways between the different sites and hence also between farms.

The on-farm biosecurity level is therefore hypothesised to be important for the prevention of *S.* Dublin between-farm transmission. Different studies have identified single factors related to local transmission associated with *S.* Dublin occurrence; however, the majority of the risk factor studies include or concern other *Salmonella* serotypes (22, 23). Furthermore, studies on *Salmonella* spp. have failed to identify specific local transmission pathways (24). This may be due to inconsistencies in the probability of transmission caused by intermittent excretion of low bacterial numbers, particularly for serotype Dublin, or the numerous possible introduction pathways (25–28). Even if the pathogen is introduced to a farm, it may not establish itself in the animals or environment if adequate internal biosecurity measures are in place.

Existing tools that employ quantitative methods to measure biosecurity or conduct risk assessment for cattle diseases have been developed (29–31). However, less attention has been paid to whether a more qualitative or semi-quantitative assessment approach, involving in-depth on-farm investigations, similar to experiences from the field of animal welfare assessment, can offer a better understanding of the on-farm biosecurity and the risk of disease introduction and establishment. A semi-quantitative Biosecurity Assessment Framework (BAF-SD) aimed at the introduction and establishment of *S*. Dublin was developed and described by Pedersen et al. (23). It comes with an electronic tool that can assist trained biosecurity assessors in performing systematic biosecurity assessments on dairy farms by conducting on-farm observations and interviewing the farmer.

The overall purpose of the current nested case–control study was to provide new information that can be used to reduce the introduction and establishment of *S*. Dublin on dairy cattle farms. The specific objectives were to (1) describe the biosecurity levels in Danish dairy cattle farms situated in *S*. Dublin enzootic areas; (2) analyse the association between on-farm biosecurity and the risk of *S.* Dublin introduction and establishment in Danish dairy cattle farms, and (3) identify the farm sections most relevant for biosecurity improvement.

## 2 Materials and methods

### 2.1 Study design and source population

For this epidemiological study, a nested case–control study was designed. From 1 September 2021 to 31 August 2022, a delineated source population of Danish dairy cattle farms at risk of becoming test-positive for *S.* Dublin was followed (see overview of farm and business definitions in Table 1). The source population (cohort) included dairy farms with recorded *S.* Dublin-tested bulk-tank milk (SD-BTM) samples and *Salmonella* level 1 (most likely free from *S.* Dublin infection) during previous 2 years or more, located in enzootic *S.* Dublin areas, defined as areas within a 10-km radius around the farm with at least one test-positive neighbouring cattle farm (see overview of definitions of infections status of farm and areas in Table 1).

**Table 1:** Definitions of cattle farm and business, infection status, and areas.

Term	Definition
Farm	Property located at a specific geographical location identified by a unique number in the Danish Central Husbandry Register (CHR). The farm may include one or more cattle herds with the same or different owners.
Business	One or more herds with the same owner at one or more farms.
*Salmonella* Dublin (*S*. Dublin) bulk tank milk (SD-BTM)	Bulk tank milk from dairy farms was tested in an indirect in-house Enzyme-Linked Immunosorbent Assay (ELISA) *Salmonella* serogroup D test, measured as a corrected test optical density coefficient (ODC%). Dairy cattle farms are tested quarterly according to the Danish National Surveillance Programme.
*Salmonella* level 1	Farms are most likely free from *S.* Dublin infection based on antibody surveillance and trade classification. Surveillance: Level 1 criteria for dairy cattle farms are (i) an average SD-BTM ELISA threshold below 25 ODC% for the last four SD-BTM samples with more than 21 days between sampling, and (ii) a maximum increase in percentage point of 20 between the last SD-BTM sample and the average of the previous three (denoted “the jump criterium”). Non-dairy farms level-1 criteria include ELISA *Salmonella* serogroup D test results below 50 ODC% in blood samples. Level-giving surveillance samples include automatically collected samples from slaughter animals by quarterly designation of farms in the Central Danish Cattle Database or the annual blood sampling of 8 or 16 animals in heifer-replacement farms. Trade classification: When cattle are moved from level-1 farms to other cattle farms, it has no effect on the *Salmonella* levels of the farms.
Enzootic *S.* Dublin area	An area of 10-km radius with at least one test-positive neighbouring cattle farm (all types of cattle farms)
Test-positive neighbour cattle farm	Cattle farm with 182 interrupted or connected days or more in *Salmonella* level 2 and a positive indirect ELISA *Salmonella* serogroup D test. Both criteria were fulfilled during the last year prior to the monthly recalculated cohort. A positive antibody test was defined as either a bulk-tank milk reaction of ≥25 ODC% or a serological sample ≥50 ODC%, regardless of monitoring purpose.
*Salmonella* level 2	Farms most likely infected with *S.* Dublin based on antibody surveillance, detected salmonellosis, or trade classification. Surveillance: Farms exceed the threshold of level-1 criteria, including a follow-up SD-BTM sample with the same ODC% threshold in dairy cattle farms. Salmonellosis: Farms with clinical signs and positive bacteriological samples for *S.* Dublin. Trade classification: Ingoing animals from farms with unknown or level-2 status (trade from a level-2 farm is only allowed if the receiving farm is part of the same business or if all animals at the receiving farm is from the same farm).
Local infection pressure	The mean number of cattle during the last year across all test-positive neighbour cattle farms within the *S.* Dublin-enzootic area.

**Figure 1:**
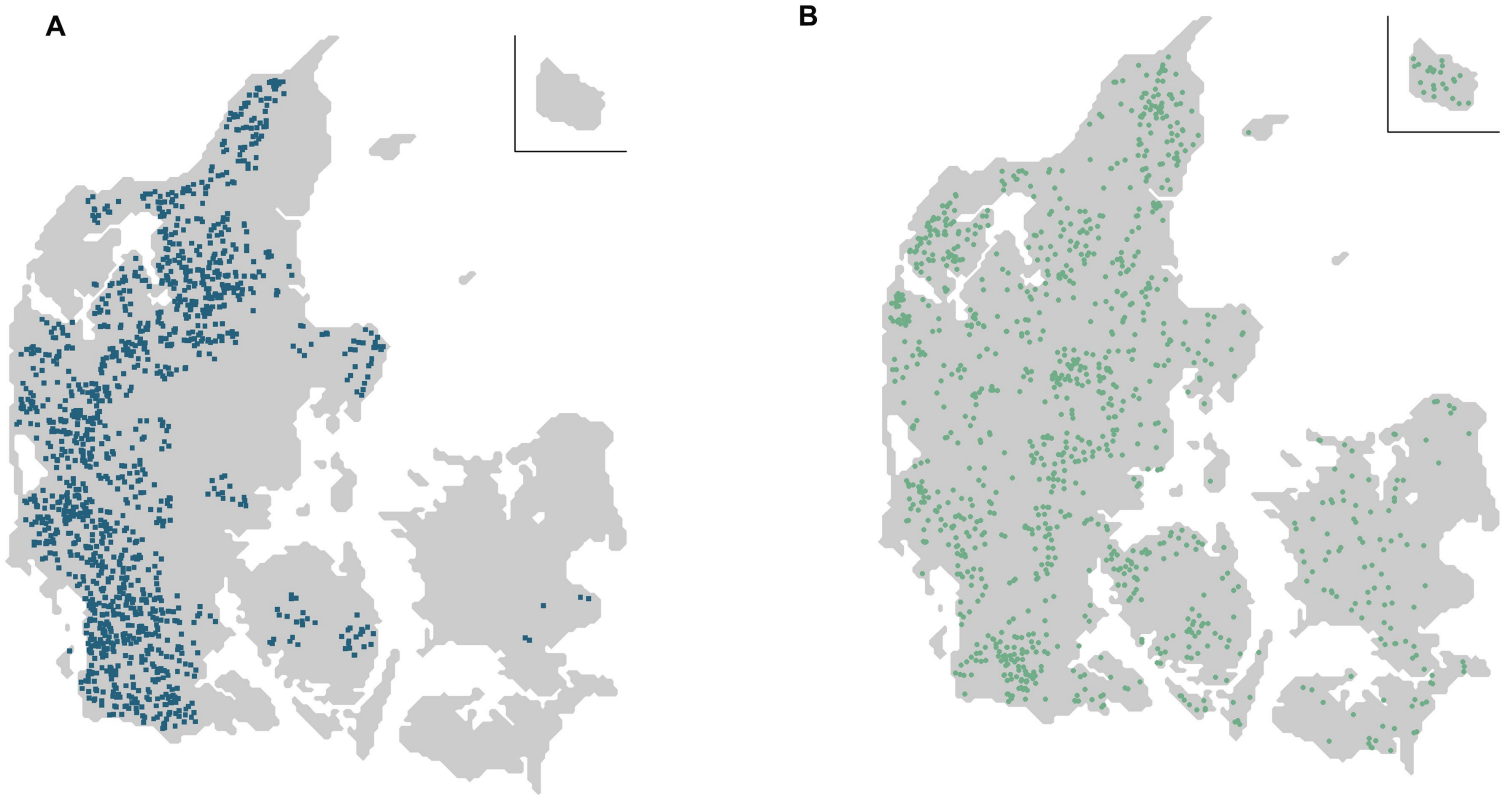
**(A)** Of the total number of 2,513 Danish dairy cattle farms at study started on 1 September 2021, the source population included 1,383 dairy farms at risk (blue squares), located in enzootic *Salmonella* Dublin areas of a 10-km radius around each farm. **(B)** Of the remaining 1,130 dairy cattle farms (green dots), 744 were located in non-enzootic areas of a 10-km radius, and 386 were in level 2 (likely infected with *S.* Dublin) or had been in level 2 within the last 2 years. Note that the maps only show dairy farms. However, dairy farms at risk (blue squares) could also be located in an *S.* Dublin-enzootic area, if a non-dairy cattle farm (not shown in the figure) fulfilled the criteria for a test-positive neighbour cattle farm.

The source population was recalculated every month to account for the new dairy farms at risk and included 1 September 2021, at study start, 1,383 of the 2,513 Danish dairy farms (Figure 1A). Dairy farms outside the source population included 744 farms in non-enzootic areas, and 386 were farms in *Salmonella* level 2 (likely-infected with *S.* Dublin) or were farms with a history of level 2 within the last two years (Figure 1B).

### 2.2 Detection of case and control dairy cattle farms in the source population

Every day during the study period, case farms were designated from the Danish Cattle Database (DCD) when they changed from level 1 to exceed the thresholds in SD-BTM, leading to *S*. Dublin level 2 in the Danish surveillance system (17). Simultaneously, a list of 20 relevant control farms (remaining test negative) were randomly selected, for each case farm, matched by the herd size groups (<100, 100–200, 200–300, 300–500, and >500 cows, measured as mean number of cows during the last year prior to study farm designation) to account for frequency of potential exposure occurrences (see overview of outcome and explanatory variables in Table 2). The first author invited farmers by phone subsequent to the designation. If case farms agreed to participate, matching control farms were contacted from the top of the control list until two controls were included per case. A case–control ratio of 1:2 was decided to accomplish more observations, within the limitation of a 1-year study period and financial resources. A visit date was scheduled respecting restrictions related to farm disease status and farmers’ availability. Farms could only be included as controls once, and they had to remain test negative in the same year and quarter of SD-BTM surveillance.

**Table 2:** Overview of outcome and explanatory variables, scale, and data origin.

Role of variable in model	Variable	Scale	Data origin
Outcome	Likely infected in the national surveillance programme for *Salmonella* Dublin (*S*. Dublin) due to exceeding the threshold in SD-BTM	Qualitative, dichotomous	Surveillance register data from the Danish Cattle Database
Primary explanatory	Individual biosecurity sections (up to 12 sections)	Quantitative, pseudo continuous	Farm observations and interviews through BAF-SD
	Overall biosecurity score, weighted		
Secondary explanatory	Animals on the pasture	Qualitative, dichotomous	
	Production type, organic		Online register data from the Danish Agricultural Agency homepage
	Ingoing animal movement		Register data from the Danish Cattle Database
	Local infection pressure	Quantitative, continuous	
	Business network	Qualitative, ordinal	

### 2.3 On-farm data collection

To collect the primary explanatory variables, information on biosecurity was obtained at a single farm visit by the same trained biosecurity assessor (the first author) using the published BAF-SD (23). Through the 7-step BAF-SD process, a single weighted semi-quantitative score of the farm biosecurity level was obtained for the 1-year risk period prior to the designation of the study farm, serving as a primary explanatory variable. As part of that process, up to 12 biosecurity sections were evaluated on each farm based on information from 56 observations and 109 interview questions posed in an open conversation style to the farmer or herd manager. The 12 sections are listed in Table 3. Each section and the weighted biosecurity level were reported on a scale from 0 to 100, where 100 represented excellent performance of all aspects of biosecurity with only a few minor deficiencies, and 0 represented the worst performance with no or the very minimum level of biosecurity barriers in place. Additionally, regarding the biosecurity section about animals on pasture, the answer was dichotomised into “animals on the pasture” or “no animals on the pasture” as a secondary explanatory variable. During the farm visit, venous blood was randomly sampled from calves between 100 and 180 days for Enzyme-Linked Immunosorbent Assay (ELISA) *Salmonella* serogroup D serological testing at Eurofins Steins laboratory, Department of Milk Testing Denmark, section for serology in Vejen, using the same test as for the surveillance programme (32).

**Table 3:** The 12 biosecurity sections included in the Biosecurity Assessment Framework for *Salmonella* Dublin introduction and establishment in dairy cattle farms (BAF-SD), published by Pedersen et al. (23).

Number	Biosecurity section	Number	Biosecurity section
1	Entrance	7	Manure
2	Pick-up-delivery calves	8	Storage of feed and feeding
3	Pick-up-delivery adults	9	Washing facilities
4	Calving facilities	10	Animals on pasture
5	Calves < 130 days	11	Vermin control
6	Cattle > 130 days	12	Carcass disposal

### 2.4 Register data collection

Data for secondary explanatory variables were obtained from the interviews, the DCD, and the Danish Agricultural Agency (DAA). Coordinates from the Geographical Information System (GIS), animal and farm records from the Central Husbandry Register (CHR) and *Salmonella* surveillance data was extracted from the DCD for the individual study farm risk period to include proxies for: (i) Local infection pressure, (ii) Business network, and (iii) Ingoing animal movement. The “local infection pressure” was defined as the mean number of cattle during the last year across all test-positive neighbour cattle farms within the enzootic area of the individual study farm (see Table 1). To account for indirect and direct transmission pathways due to multisite business, we identified cattle herds and farms in Denmark with the same ownership as the study unit farm and categorised the number of farms including cattle herds belonging to each study farm as a proxy for “business network.” Additionally, records of animal movement were combined with the study unit business network information and movement of animals from farms outside to inside the business network, including shows and/or common pasture areas, and dichotomised into a proxy for “ingoing animal movement.” Publicly available farm information about ownership and production type, that is, organic or conventional, was downloaded from the DAAs homepage on 3 October 2022 (33).

### 2.5 Establishment of dataset

The data management was performed in the statistical software R version 3.6.1 (34): (i) the daily information of potential case and control farms were shared with the first author per email in-house at the SEGES Innovation company (SEGES Innovation P/S, Aarhus, Denmark) during the study period, (ii) processed data for secondary explanatory variables was organised into Microsoft Excel (Microsoft Corporation, Redmond, WA, United States) spreadsheets, and (iii) merged with BAF-SD data collected on-farm on paper and entered into a final Microsoft Excel spreadsheet for analysis together with information about production type.

### 2.6 Data control and descriptive data analysis

The first author performed the management of BAF-SD data and manually checked for errors by a controller. Using descriptive statistics in the statistical software R version 3.6.1: (i) we carefully inspected data and outliers for incorrect data entry by measures including frequency distribution, summary statistics report, and compared the source and sampled population, (ii) we described explanatory variables by measures including cross-tabulation, standard deviations, percentiles, and graphical illustrations including scatterplots, box-plots, histograms, and violin plots.

### 2.7 Statistical analysis

Pairs of explanatory variables were initially checked for collinearity (*p* < 0.05) using Fisher’s exact test, Spearman’s rank correlation coefficient, and point–biserial correlation. If collinearity between pairs of explanatory variables was observed, the one with the highest *p*-value in the univariable analysis was excluded from the subsequent multivariable modelling.

Thereafter, we analysed data using the R-survival package, in statistical software R version 4.3.1 (35) for conditional logistic regression models stratified by matching pairs with disease status (case or control) as outcome. Initially, we tested the univariable association between each explanatory variable, including the semi-quantitative biosecurity assessment for each biosecurity section (one by one), and the outcome. Thereafter, we included the overall weighted biosecurity score and all secondary explanatory variables and possible meaningful interactions in a multivariable model (Equation 1), which is expressed as


(1) $$\text{logit}(P(Y_{\mathrm{ij}}=1))=\beta_{A}A_{\mathrm{ij}}+\beta_{B}B_{\mathrm{ij}}+\beta_{C}C_{\mathrm{ij}}+\beta_{d}d_{\mathrm{ij}}+\beta_{e}e_{\mathrm{ij}}+\mu_{\mathrm{Fj}}$$

where $P(Y_{\mathrm{ij}}=1)$ represents the probability that a farm 𝑖 in stratum 𝑗 is a case; *A*, *B*, and *C* represent the categorical variables “production type, organic,” “business network,” and “ingoing animal movement”; *d* and *e* represent the numerical variables “overall biosecurity score, weighted” and “local infection pressure”; $\mu_{\mathrm{Fj}}$ is the random intercept for farm size group *F_j_*, with $\mu_{\mathrm{Fj}}$ *∼ N (0,σ2).* The model is stratified by matching pairs *j*, controlling for confounding at the stratum level.

The model was manually fitted by backward stepwise elimination. Akaike’s Information Criteria was used as an elimination criterion and a likelihood-ratio test to evaluate the explanatory variables criterion for inclusion (36). Furthermore, all excluded explanatory variables were reintroduced one by one to the final model to check for overlooked statistical associations with the outcome and to consider possible confounding defined by more than 20% change in final model estimates when the variables were reintroduced.

### 2.8 Ethics approval and consent to participate

At the beginning of each farm visit, the objective of the study was repeated for the farmer, and written approval with consent to contribute to data collection using the BAF-SD and to grant access to farm data from registers was obtained from the farmers. It was clear that only anonymised data and results would be made publicly available, and that farmers could withdraw from the study at any time. The study was ethically approved by the institutional Research Ethics Committee of Science and Health at the University of Copenhagen, case number: 504-0306/22-5,000. The National Committee for the Protection of Animals approved the sampling of venous blood from animals, permit number: 2021-15-0201-00946.

## 3 Results

### 3.1 Population

In total, 37 case and 74 control farms were included in the study, including two case farms testing serologically positive above threshold (≥50 optical density coefficient % [ODC%]) in individual animals immediately before the farm visit and repeatedly being test-positive in random sampling during the farm visit. Ninety-nine included farms (89%) were designated in the third and fourth quarter of 2021, and the remaining 12 farms (11%) in the first and second quarter of 2022. All case farms and 69 control farms were located on the Jutland Peninsula; the remaining control farms were on the islands of Funen and Zealand. Forty farms with a median herd size of 127 cows declined to participate, including five case farms (Table 4), mainly due to lack of time, interest, or the farmer being close to retirement (10 out of the 40 farms were either closed or had stopped milk production within 3 years after designation). Additionally, to the 40 farms declining to participate; (i) it was not possible to establish contact to one case farm and 10 control farms, (ii) fourteen control farms was excluded due to other reasons (recent stop in milk production, no calves, etc.), and (iii) another two case farms were excluded due to a change in ownership leading to a lack of ability to respond to the interview part about the previous year of practices, and due to suspected cross-reactions upon positive culture for *Salmonella* Typhimurium, respectively. None of the tested calves in control farms were serologically positive above the ELISA *Salmonella* serogroup D threshold (≥50 ODC%). In 25 out of 37 case farms, at least one calf was serologically positive. Visits to case farms were prioritised to have their biosecurity level assessed close to the outcome of becoming test-positive, and 87.5% were visited within 20 days of confirmed level-2 status. Comparing study farms with our source population at study start, the median herd size was higher among study farms (Table 4). In comparison, farms declining to participate had a smaller median herd size. Additionally, the median number of test-positive neighbour farms was similar among the source population and control farms but higher for case farms.

**Table 4:** Mean, median, p10 = 10% (percentile), and p90 = 90% (percentile) herd size and number of test-positive neighbour cattle farms within the enzootic *Salmonella* Dublin area for source, sampled, and declining to participate populations.

Entity	Source population				Sampled population								Declined to participate							
					Case				Control				Case				Control			
Number of farms	1,383				37				74				5				35			
Measures	Mean	Median	p10	p90	Mean	Median	p10	p90	Mean	Median	p10	p90	Mean	Median	p10	p90	Mean	Median	p10	p90
Herd size	214	172	65	405	244	198	85	518	257	191	85	548	159	138	119	214	200	124	45	511
Test-positive neighbour farms within the enzootic *Salmonella* Dublin area	7	4	1	15	11	9	2	21	7	4	1	16	12	12	7	18	5	5	1	10

Herd size is measured as the mean number of cows during the last year, and test-positive neighbour cattle farms within the enzootic *S.* Dublin area are defined in Table 1.

### 3.2 Descriptive statistics and univariable analysis for biosecurity assessment

All 12 biosecurity sections were assessed in 23 case farms; 9 farms did not have animals on the pasture, and 3 did not move calves aged below 4 months off the farm. One farm had calving facilities, and another had storage for feed at cooperating farm properties. For control farms, all biosecurity sections were assessed in 44 farms, 26 farms did not have animals on the pasture, and 4 did not move calves aged below 4 months off the farm.

**Figure 2:**
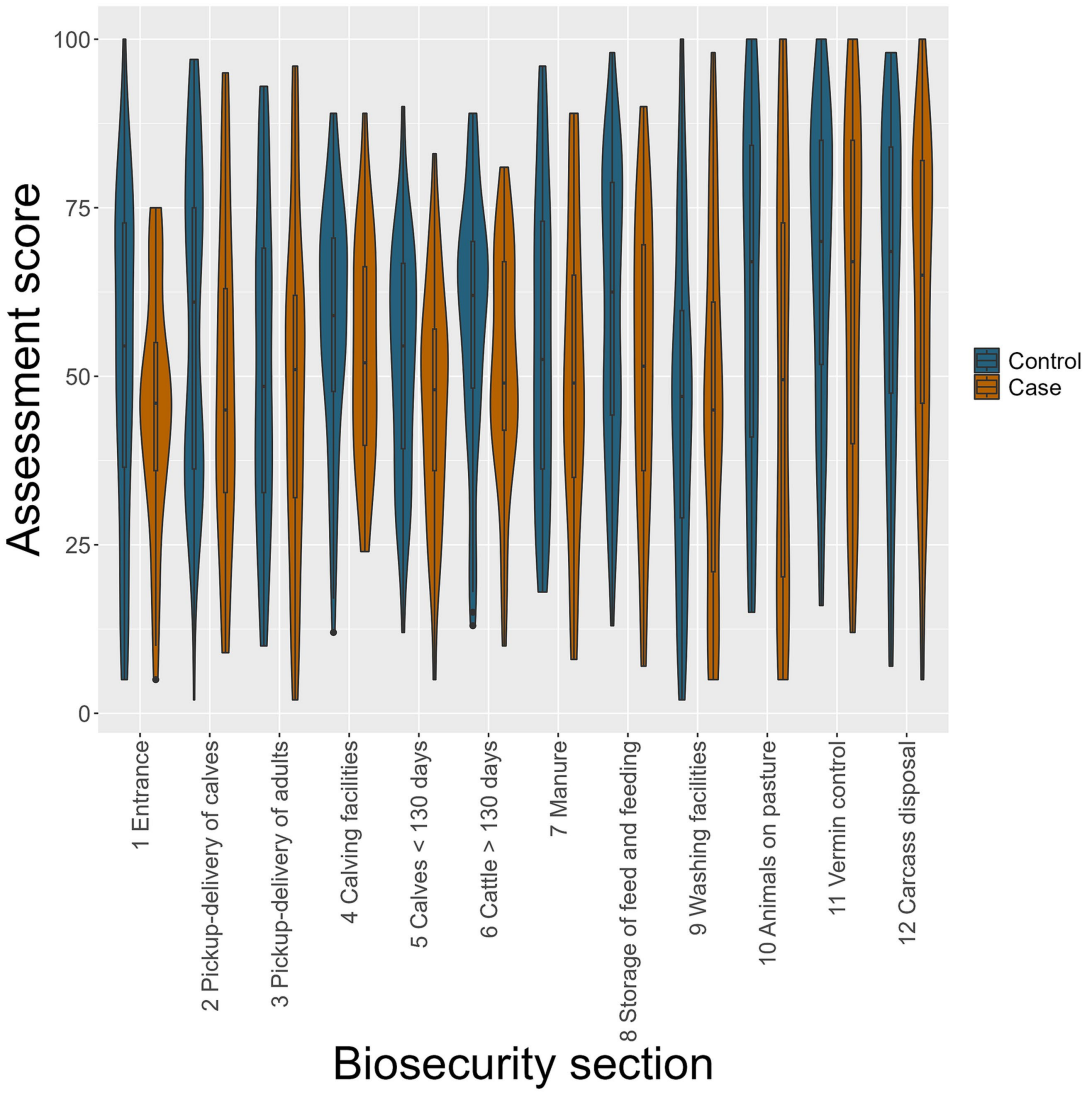
Violin and box plots of up to 12 assessed biosecurity sections in 74 control (blue) and 37 case (brown) farms using the Biosecurity Assessment Framework for *Salmonella* Dublin. Scoring from 0–100, 0 is a total lack of biosecurity measures, and 100 is excellent biosecurity.

Comparing mean biosecurity scores for each biosecurity section between cases and controls, a lower mean score was obtained in all 12 sections among case farms. Notably, the median biosecurity score was below 50 (below which it would be interpreted as not acceptable biosecurity); in seven out of 12 sections for case farms and 2 sections for control farms (Figure 2). The initial univariable analyses for each of the 12 biosecurity sections showed that the odds of having received higher section biosecurity scores were, with 90% confidence interval (CI; *p* < 0.1), higher for control farms than for case farms for the following five sections: “1 entrance area,” “2 pick-up-delivery of calves,” “5 calves < 130 days,” “6 cattle >130 days,” and “8 storage of feed and feeding,” leading to ORs below 1 for being a case with each 10-point increase in section biosecurity score (Table 5).

**Table 5:** Results of conditional univariable logistic analysis of biosecurity assessment and secondary explanatory variables in a nested case–control study with 37 case farms and 74 control farms matched by herd size groups.

Univariable analysis	Conditional logistic regression strata (37 cases and 74 controls)							
Variable	Level	Case	Control	OR	95% CI		SE	*p*-value
1 Entrance	(Count. increments of 10)	37	74	0.84	0.69	1.02	0.10	0.08
2 Pickup-delivery of calves		34	70	0.84	0.69	1.03	0.10	0.08
3 Pickup-delivery of adults		37	74	0.93	0.78	1.12	0.09	0.44
4 Calving facilities		36	74	0.84	0.64	1.09	0.13	0.17
5 Calves < 130 days		37	74	0.80	0.62	1.03	0.13	0.07
6 Cattle > 130 days		37	74	0.81	0.64	1.02	0.12	0.07
7 Manure		37	74	0.92	0.76	1.10	0.09	0.35
8 Storage of feed and feeding		36	74	0.83	0.68	1.02	0.10	0.07
9 Washing facilities		37	74	0.97	0.83	1.14	0.08	0.74
10 Animals on pasture		28	48	0.89	0.74	1.08	0.10	0.23
11 Vermin control		37	74	0.89	0.74	1.08	0.10	0.25
12 Carcass disposal		37	74	0.96	0.82	1.13	0.08	0.63
Overall biosecurity score, weighted		37	74	0.63	0.43	0.92	0.20	0.009
Local infection pressure	(Count. increments of 1,000)	37	74	1.14	1.03	1.27	0.05	0.008
Business network	1	19	46	Ref.				
	2	11	17	1.69	0.65	4.44	0.49	0.47
	≥3	7	11	1.96	0.52	7.34	0.67	
Ingoing animal movement	No	27	46	Ref.				
	Yes	10	28	0.57	0.23	1.43	0.47	0.23
Production type, organic	No	27	62	Ref.				
	Yes	10	12	1.77	0.72	4.31	0.46	0.22
Animal on the pasture	No	9	26	Ref.				
	Yes	28	48	1.72	0.69	4.25	0.46	0.23

The odds ratio (OR), 95% confidence interval (95% CI), standard error (SE), and significance level (*P*) are given for each explanatory variable. Unscored biosecurity sections in farms with fewer than 12 sections were excluded from the univariable analysis.

**Figure 3:**
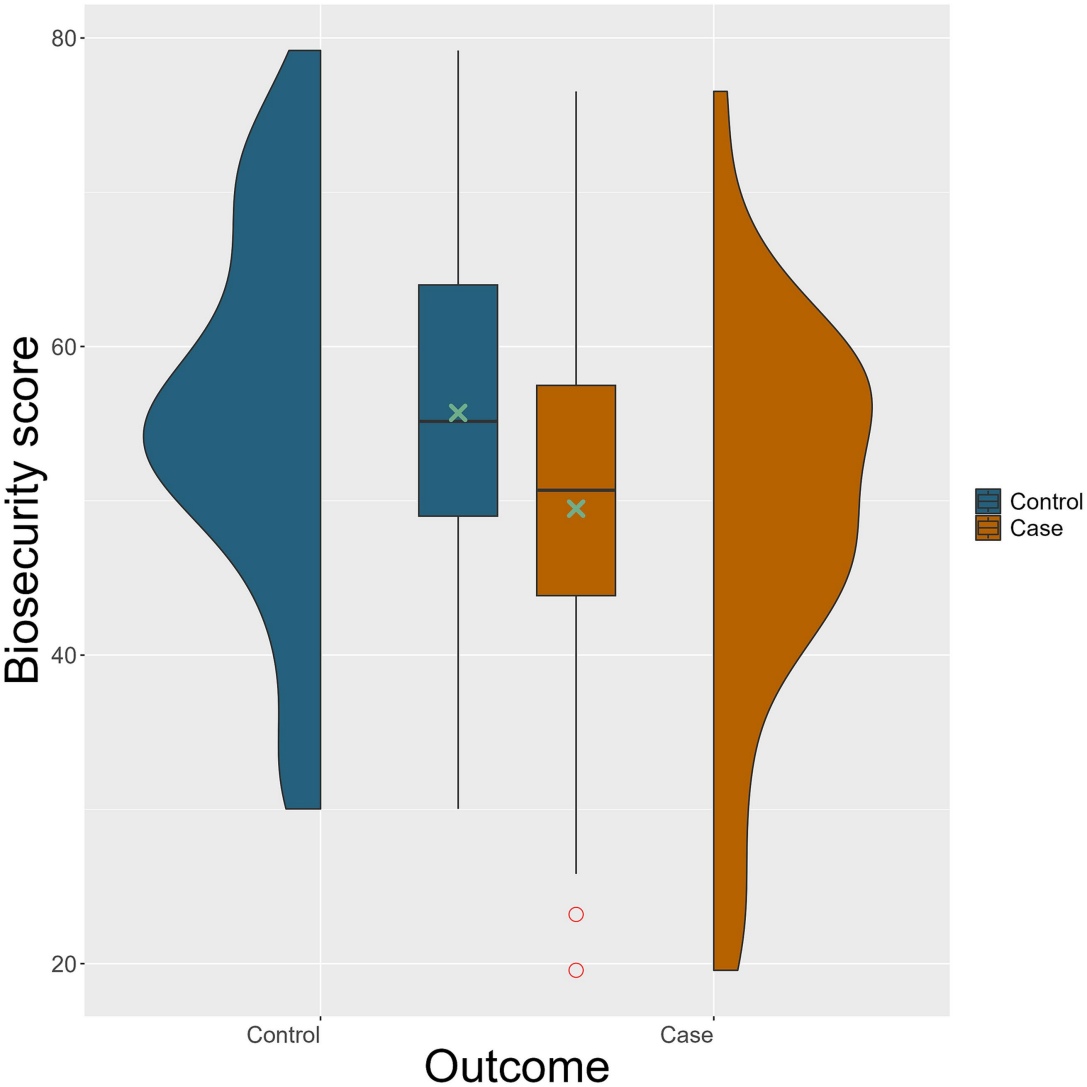
Violin and box plots of weighted overall biosecurity score for the 74 control (blue) and 37 case (brown) farms. Green cross (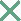) indicates the mean biosecurity score for cases and controls, respectively, and circle (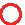) indicates outliers. Weighted biosecurity assessment scores can range from 0 to 100, where 0 is a total lack of biosecurity measures and 100 is excellent.

The weighted biosecurity scores for the 111 included farms are illustrated in Figure 3. Among case farms, the highest weighted biosecurity score was 76.5, the lowest was 19.6 out of 100 points, and the mean score was 49.5. For control farms, the highest score was 79.2, the lowest was 30.0, and the mean was 55.7. In the conditional univariable analysis, the odds were significantly lower (odds ratio [OR] = 0.63, *p* = 0.009) for each 10 weighted biosecurity score increase to be a case farm rather than a control farm indicating an association between reducing biosecurity level and a risk of becoming test-positive for *S*. Dublin (Table 5).

### 3.3 Descriptive statistics and univariable analysis for secondary explanatory variables

**Figure 4:**
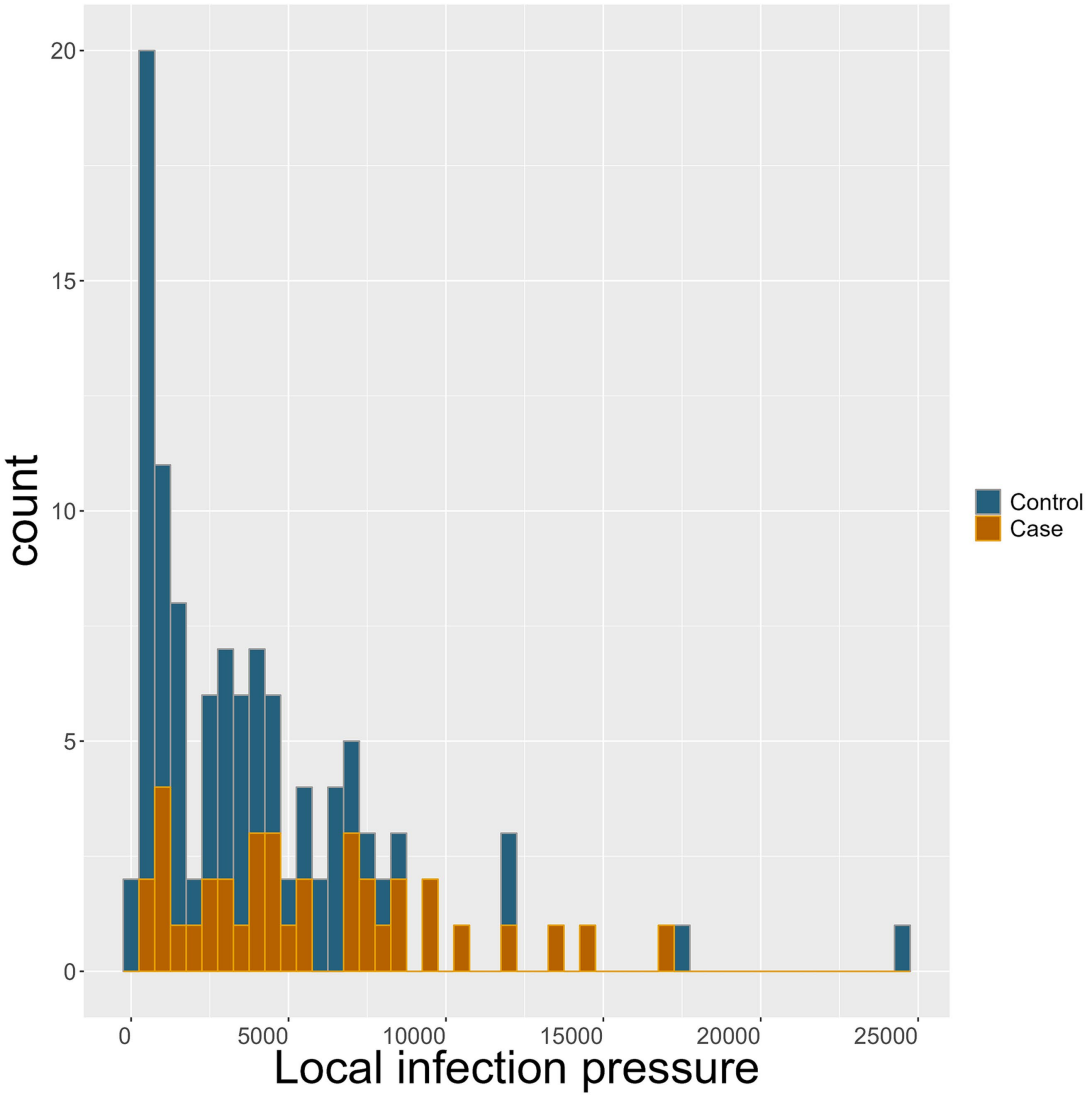
Local infection pressure: the mean number of cattle in all *Salmonella* Dublin test-positive neighbour cattle farms within the enzootic *S.* Dublin area of 10 km around the individual study farm during the last year prior to the month of designation for control (blue) and case (brown) farms.

Becoming a case farm was associated with higher odds of having increased local infection pressure. The OR of becoming a case was 1.14 (95% CI: 1.03–1.27, *p* = 0.008) for each 1,000 head increase in the number of cattle present in test-positive cattle farms in a 10-km area around the farm. This should be seen in the light of the extensive range from 83 to 24,639 cattle in neighbouring test-positive farms in the available dataset, illustrated in Figure 4.

Among the controls, a higher proportion (62%) of the businesses consisted of a single farm in the business network compared to cases (51%). In general, 34% of the farms had animals moved to the business network within a year, with a higher percentage (38%) among controls (i.e., 28 of 74 farms) compared to 27% of the cases (10 of 37 farms). Moreover, 16% of the control farms and 27% of the case farms were certified organic producers, and 65% of the control and 76% of the case farms had animals on the pasture during the grassing season. None of the last four secondary explanatory variables appeared to be associated with being a case farm in our dataset. Results of the univariable analyses are shown in Table 5.

### 3.4 Biosecurity regression model for becoming a *Salmonella* Dublin test-positive dairy farm

The results of the multivariable conditional logistic regression model are illustrated in Table 6. The final model included biosecurity level (between 19.6 and 79.2) and local infection pressure as the only significant explanatory variables. Within the same herd size group, the odds for a 10-unit increase in biosecurity level and 1,000-unit increase in local infection pressure in farms becoming test-positive relative to the odds for a 10-unit rise in biosecurity level and 1,000-unit increase in local infection pressure for farms remaining test negative were 0.64 (95% CI: 0.43–0.96, *p* = 0.03) and 1.13 (95% CI: 1.01–1.25, *p* = 0.03), respectively. This demonstrates the importance of improving the overall biosecurity level and lowering the local infection pressure for prevention against *S*. Dublin introduction and establishment in dairy farms.

**Table 6:** Significant variables for becoming *Salmonella* Dublin test-positive farms according to the final multivariable analysis comprising 37 case farms and 74 matched control farms by herd size groups in a nested study design by conditional logistic analysis method.

Multivariable analysis	Conditional logistic regression strata (37 cases and 74 controls)					
Variable	Level	OR	95% CI		SE	*p*-value
Overall biosecurity score, weighted	(Count. increments of 10)	0.64	0.43	0.96	0.20	0.03
Local infection pressure	(Count. increments of 1,000)	1.13	1.01	1.25	0.05	0.03

The odds ratio (OR), 95% confidence interval (95% CI), standard error (SE), and significance level (*P*) are given for each explanatory variable.

## 4 Discussion

In this study, we found that the overall biosecurity level is associated with the risk that dairy farms become test-positive in the ongoing Danish *S.* Dublin surveillance programme, indicating that they have become newly infected with *S*. Dublin. We assessed biosecurity practices using the published BAF-SD in dairy farms classified as newly test-positive (37 cases) and remaining test-negative (74 controls), respectively. Adjusted for the confounder local infection pressure, approximated by the total number of cattle in test-positive neighbour farms within 10 km, a 10-unit increase in biosecurity level was significantly associated (*p* < 0.05) with reduced odds (OR = 0.64) of becoming a *S.* Dublin test-positive dairy farm within the same herd size group. At the same time, none of the other tested variables were found to be associated with the outcome. While other studies have investigated environmental factors associated with the *S.* Dublin introduction and establishment (22, 23), this is, to our knowledge, the first study quantifying an association between overall measurable biosecurity level and the risk of dairy farms becoming test-positive for *S.* Dublin.

The study also provided insights into the level of biosecurity targeting *S*. Dublin in Danish dairy farms. We did not observe farms with a very good (score 80 to <90) or excellent (score of 90 or above) overall biosecurity level in any study farms. Despite a higher biosecurity level in control farms, we observed considerable room for improvement across all biosecurity sections in many farms. Similar results have been obtained in 50 dairy farms in Belgium using another available biosecurity assessment system, Biocheck^®^UGent (Biocheck.Gent BV, Dentergem, Belgium), where no farms received a score above 83 in external biosecurity and 69 in total biosecurity out of an ideal biosecurity of 100 points (29). Furthermore, the system with different classifications and scores has been used to identify an association between apparently free-BVD status and higher biosecurity score (37). The similarity is interesting. The open structure of dairy farms enables multiple introduction pathways for infectious agents; as described by others, this may add to the failure in identifying of single risk factors (24). Indeed, we did not identify clear associations between biosecurity scores for the individual biosecurity sections and risk of becoming test-positive for *S.* Dublin, although there were some indications that cases had lower scores in five sections than matched control farms. However, we identified an association with the overall level of biosecurity. One explanation for this could be the need to accumulate all risk factors, as the introduction and establishment of the bacteria can happen through many different pathways over time. Hence, it can be argued that the biosecurity of dairy farms in enzootic areas of Denmark is insufficient to resist the introduction and establishment of a local transmission-driven pathogen, such as *S.* Dublin, and potentially other infectious agents. To some extent, this might also explain the association we found between local infection pressure and the odds of becoming test-positive for *S.* Dublin, with an OR of 1.13 for each 1,000 head increase in the number of cattle in test-positive neighbour farms within a 10-km radius. Local infection pressure is a risk factor repeatedly recognised by similar proxies across countries (38–44).

### 4.1 Study limitations

Surprisingly, no interaction between the overall weighted biosecurity score and local infection pressure was identified. This raises the question of whether the used biosecurity framework can fully quantify the true biosecurity level, either because of unknown pathways for introduction and establishment not captured by the framework, assessment reliability, or other study limitations.

A poorly understood pathway might be the risk coming from wild birds. High numbers of migrating birds are recorded periodically on some Danish dairy farms, but the scientific evidence for wild birds as reservoirs or mechanical transmitters for *S.* Dublin is not clear (45–47). In the used framework, the biosecurity section 11, “Vermin control,” was given the lowest weight in the final score by the experts during the development of the framework.

Regarding consistency in assessment score, a single inter-observer reliability test indicated a moderate interclass correlation for the framework’s scores (23). To minimize the observer effect, we used the same trained assessor in all farms. However, intraobserver reliability was never tested due to lack of available resources, and due to expected dynamics in on-farm biosecurity and time needed not to be able to recall assessment scores from previous biosecurity scoring sessions. Also, the biosecurity level was assessed for 1 year prior to designation as case or control, which introduces potential for recall bias and changes over time that are very difficult to capture and quantify in this type of study.

Another limitation of the study is that, according to the Danish legislation, farmers must inform visitors about their farm’s health status, excluding a blinded study design, with possible introduction of performance bias. However, we consider that the potential bias due to the unblinded study design is negligible due to the inclusion of a scoring guide in the BAF-SD.

Because the cumulated risk combines probability and frequency, another limitation is unmeasured frequency variabilities within each herd size group. In BAF-SD, a separate section on the purchase and replacement of animals is not included but merged into sections 2, “pickup-delivery of calves,” and 3, “pickup-delivery of adults.” Additionally, the risk through animal movement was supported by whether animals had been moved to the business network from other cattle businesses, shows, and/or common pasture, limited by the possibility to include frequency and number of animal source businesses for this study. The “ingoing animal movement” variable was overrepresented among the control farms. The lack of association with the risk of becoming test-positive agrees with other studies conducted under the given national movement restriction or movement from test-negative farms in Denmark (15, 42). Interestingly, a recently published network analysis of Danish farms identified movement activity as a predictor for farms becoming classified as infected with *S.* Dublin (17), supporting studies from other countries with less strict *S*. Dublin-related movement restrictions at the time of study (44, 48). The authors of the Danish network analysis (17) suggest that the results about the strong effect of animal movements may be somewhat overestimated due to multisite business structures that were not accounted for in the model. Such movements still occur in Denmark, because animal movements between farms within the same-owner business networks are not as strictly limited as the movement of animals out of test-positive business structures for live purposes under the Danish legislation. Moreover, farm status changes may occur as an administrative consequence of risky animal movements and not always due to a change in status determined by test results. However, it is likely that *S*. Dublin survives more easily and longer by recirculation between and within multisite farms, as supported by the association with lower release hazard from *Salmonella* restriction in Swedish multisite cattle farms (49). In this study, an association between multisite businesses and the odds of becoming test-positive was not identified, despite a higher proportion of multisite business structures among the case farms. Indeed, a similar tendency was observed for production type, with 27% of the case farms certified as organic, compared to 16% among controls. Organic production has been associated with both being *Salmonella* test-positive and time to recovery, but to our knowledge, not as a risk for introducing *S.* Dublin (24, 42, 50). The authors of those studies interpreted the findings as related to the extended period of cow-calf contact before separation and stocking density among calves due to requirements by regulation in infected organic farms. In addition, collinearity was observed between animals on the pasture and organic production, but not with the overall biosecurity score. It seems logical that having animals on the pasture is the biologically plausible explanation between the two variables. However, in the multivariable analyses we only included “production type, organic” as potential interaction or confounder in favour of whether the farm had animals on the pasture, because pasture management was covered in the BAF-SD and organic farmers’ perception of the benefit of biosecurity measures has been measured as lower compared to conventional farmers (51).

Single biosecurity sections were not identified as significant risk factors in this study. An explanation may be that type-II errors are introduced due to a small sample size. In the univariable analyses, five biosecurity sections were significant (at a 90% confidence interval) with the outcome of becoming test-positive (section 1 “entrance,” 2 “pick-up-delivery of calves,” 5 “calves < 130 days,” 6 “cattle > 130 days,” and 8 “storage of feed and feeding”). In general, the number of professional visitors and visitors in contact with animals is higher in cattle farms than in farms with other livestock animals, and with an increasing number with herd size (52). Nevertheless, cattle farms often lack proper entrance biosecurity measures (53–56). Indeed, different single risk factors related to the entrance area, such as the use of protective clothing and a clean parking area for visitors, have been associated with the risk of *Salmonella* introduction (57, 58). Similar to other Scandinavian countries, a separate loading area for animals is not available in many Danish cattle farms, even though *Salmonella* bacteria can be cultured from livestock transport vehicles for cattle (53, 54, 59, 60). Segregation of the haulier and the livestock transport vehicles from the internal farming area is weighted high in the BAF-SD scoring guide for the section 2 “pickup-delivery of calves,” and the tendency toward a significant association with the odds of becoming test-positive is therefore not surprising. Calves are the most susceptible and infectious age group, and a similar tendency was not observed for the biosecurity section 3 “pickup-delivery of adults,” even though older animals were often picked up near feeding tables and thereby potentially led to contamination of feed. Other studies have found that open storage of silage and concentrate is associated with dairy farms being positive for *Salmonella* spp. (58, 61), supporting the findings for the biosecurity section 8 “storage of feed and feeding’ in this study. *S.* Dublin is rarely isolated from feed, and the pH value in ensiled forage does not promote the survival of *Salmonella* (62, 63). Most likely, the correlation is linked to contamination of the farm’s feed. Certainly, this introduction and establishment pathway is not unthinkable. Modern feeding procedures involve storage of feed in open silos often located close to contaminated transport driveways or on-farm washing facilities. Moreover, feeding practices related to the total mixed rations may lead to close contact between the equipment, feed, and animals. Therefore, an oral-faecal transmitted pathogen, such as *S.* Dublin, can rapidly be established from point contamination to many animals within the farm. The biosecurity section 9 “washing facilities” was not found associated with the risk of becoming a case farm. This might be explained by an inadequate level of biosecurity in the majority of both case and control farms for this section.

Nonetheless, the results need to be considered with precautions due to both the sample size in this study and the limitations in the accuracy of the BTM test programme. The surveillance has been evaluated with an estimated herd sensitivity, specificity, positive predictive value (PPV), and negative predictive value (NPV) at 15% true herd-level infection prevalence of ~0.95, ~0.96, ~0.80, and 0.99, respectively (64). We improved the accuracy by including dairy farms from source population testing serologically positive on calves between 3 and 6 months, and furthermore testing a randomized sample of calves between 100 and 180 days at farm visit in all study units (65). All control farms were ELISA-negative (<50 ODC%) in blood-sampled calves. However, calves in 12 out of 37 case farms were also serologically negative on the blood samples, which could indicate either recent disease introduction in other barn sections than where the calves are housed, misclassification, or strong segregation and control measures in place to protect the calves at the farm level. Furthermore, graphic evaluation of 5-year BTM profiles of study farms indicates that some farms were misclassified as newly test-positive, but could have been reactivated infection with two years of low serological values on BTM, delayed responses in BTM reactions, or negative follow-up ELISA BTM testing indicating false-positive reaction in the first sample, while some control farms likely were in a recovery period where latent infection could not be completely ruled out.

In conclusion, this study could not identify single biosecurity sections as clear risk factors for the introduction and establishment of *S*. Dublin in Danish dairy farms. Still, the overall expert-weighted biosecurity score was significantly lower in dairy cattle farms that became *S.* Dublin test-positive than in herd size-matched control farms that remained test-negative. Hence, after being adjusted for local infection pressure in a multivariable statistical model, the overall biosecurity level is deemed to have a preventative effect against the introduction and establishment of *S*. Dublin. Moreover, we can conclude that the current level of biosecurity is insufficient to resist the infection pressure from the surroundings. Under the current biosecurity levels, the local infection pressure needs to be reduced to lower the number of new test-positive dairy farms in Denmark. The study illustrates the complicated relationship between infection pressures, biosecurity, and farming practices and structures in intensive dairy farming today.

## Data Availability

The datasets presented in this article are not readily available because we do not have the authority to share the data used for analysis. Requests to access the datasets should be directed to larp@seges.dk.
